# Impact of combined exercise on blood DNA methylation and physical health in older women with obesity

**DOI:** 10.1371/journal.pone.0315250

**Published:** 2024-12-16

**Authors:** Atchara Dawangpa, Pitaksin Chitta, Guilherme da Silva Rodrigues, Nutta Iadsee, Natália Y. Noronha, Carla B. Nonino, Carlos R. Bueno Júnior, Chanachai Sae-Lee

**Affiliations:** 1 Research Division, Faculty of Medicine, Siriraj Hospital, Mahidol University, Bangkok, Thailand; 2 Department of Clinical Pathology, Faculty of Medicine Siriraj Hospital, Mahidol University, Bangkok, Thailand; 3 Department of Internal Medicine, Ribeirão Preto Medical School, University of São Paulo, São Paulo, Brazil; 4 Health Sciences Department, Ribeirão Preto Medical School, University of São Paulo, São Paulo, Brazil; 5 School of Physical Education and Sport of Ribeirão Preto, University of Sao Paulo, Sao Paulo, Brazil; Shanghai Jiao Tong University School of Medicine, CHINA

## Abstract

This study examined the effects of a 14-week combined exercise program on blood DNA methylation (DNAm) and its potential biological pathways in normal-weight, overweight, and obese older women. A total of 41 participants were assessed at baseline, 7 weeks, and 14 weeks into the training. Their whole-blood DNAm profiles were measured using the Infinitum MethylationEPIC BeadChip, alongside physical and biochemical health evaluations. The results showed notable health improvements, with decreases in blood pressure and cholesterol levels in the overweight and obese groups. Blood triglycerides were reduced only in the overweight group. Physical performance also improved across all groups. At 14 weeks, 1,043 differentially methylated positions (DMPs) were identified, affecting 744 genes. The genes were linked to biological processes, such as cellular metabolism, with significant pathway enrichment related to oxidative phosphorylation and chemical carcinogenesis. Additionally, the overweight group experienced significant reductions in methylation levels at eight lipogenesis-related genes. Protein EpiScore analysis revealed decreased levels of CCL11, VEGFA, and NTRK3 proteins at 14 weeks compared to baseline. Despite these significant molecular changes, there was no observable difference in DNAm age after the intervention. This study highlights how combined exercise can modify DNAm patterns in older women, particularly in lipogenesis-related genes, but suggests that further research is needed to understand the full implications for biological ageing.

## Introduction

As of 2014, the World Health Organization (WHO) estimated that there were more than 1.9 billion overweight adults worldwide, of whom over 600 million were classified as obese [1]. Obesity is a metabolic disorder that results from an imbalance between energy intake and expenditure. According to the WHO, being overweight is a significant risk factor for non-communicable diseases such as type 2 diabetes, endocrine disorders, cardiovascular disease, and certain types of cancer [2–4]. These chronic conditions lead to premature death and impose a heavy burden on healthcare systems and economies worldwide [5, 6].

It is widely recognised that exercise can have profound effects on all physiological systems, improving performance and promoting better health through sub-cellular changes [7]. In recent years, significant progress has been made in understanding the cellular and molecular benefits of exercise. Epigenetic modification plays a crucial role in regulating transcription, and one of the major modifications is DNA methylation (DNAm), which is responsible for governing chromatin structure and controlling gene expression [8]. Generally, DNAm is a transfer of a methyl group into the 5-carbon of cytosine to form 5-methylcytosine, which enables heritable changes in gene expression without changes in DNA sequence. Recent evidence suggests that a significant proportion of the beneficial effects of exercise is linked to epigenetic alterations, which modify the expression of various genes related to both physiological and pathological conditions [9].

Furthermore, a six-month intervention in healthy men has shown that exercise can induce genome-wide alterations in DNAm in adipose tissues, potentially affecting adipocyte metabolism [10]. Additionally, the expression of the benefits of exercise in pre-diabetes women, such as changing the methylation patterns of genes related to energy metabolism, cell differentiation, and tumour suppression, can be found in blood [11]. Recent studies suggest a link between global DNA methylation and altered lipid metabolism. Abnormal methylation at the CpG sites of the *ABCG1* and *PHGDH* genes impairs lipid metabolism, leading to severe complications in metabolic disorders such as obesity and myocardial infection [12, 13]. Additionally, altered methylation patterns in the promoter regions of genes encoding key lipid metabolism enzymes (*FASN*, *SCD*, *ACC*) are noted in various metabolic states [14]. Regular exercise also enhances the expression of genes involved in lipid uptake and utilisation in skeletal muscle [15].

Genome-wide DNAm in different biological specimens of obesity have been identified specifically in blood samples [16]. Moreover, increased BMI in individuals has been associated with stronger methylation of genes related to hypoxia-inducible transcription factor pathways, both in blood cells and in adipose tissue [17]. Nevertheless, the alterations in blood DNAm of obese individuals after physical activity remain poorly described. Moreover, the understanding of the connection between exercise and DNAm in the context of obesity can have significant public health implications. It can help design exercise guidelines tailored to individuals with overweight or obesity, guiding the development of targeted interventions, and contributing to the growing body of knowledge on the role of epigenetic modifications in health and disease. Therefore, the current study aimed to 1) investigate the effect of combined exercise in blood DNAm of overweight and obese older women; 2) explore the potential biological pathways associated with combined exercises; and 3) investigate the DNAm age after combined exercise.

## Materials and methods

### Ethic approval and participants

The participants in this study were sub-grouped from the research (a randomised controlled clinical trial) conducted by da Silva Rodrigues and Noronha [9, 11]. This study received ethical approval from the Human Research Ethics Committee of the Ribeirão Preto School of Physical Education and Sport, University of São Paulo (EEFERP-USP), with registration number CAAE: 9582817.0.0000.5656. Furthermore, it was duly registered with the Brazilian Registry of Clinical Trials under the identifier RBR-3g38dx (15/05/2018). The date of the first enrolment was 10/02/2018 and the date of the last enrolment was 17/07/2019. All methods in this study were performed in accordance with the relevant ethical guidelines and regulations. All research protocols were conducted in full compliance with the ethical standards outlined in the Declaration of Helsinki. Participants were required to fast overnight before sample collection, and fasting blood samples were drawn by trained medical personnel under strict sterile conditions. Participants were fully informed about the study’s purpose, risks, and procedures, and gave written informed consent before participating. The required sample size was calculated using G*Power [18], based on a *t*-test for means with the statistical power set at 0.903.

**Fig 1:**
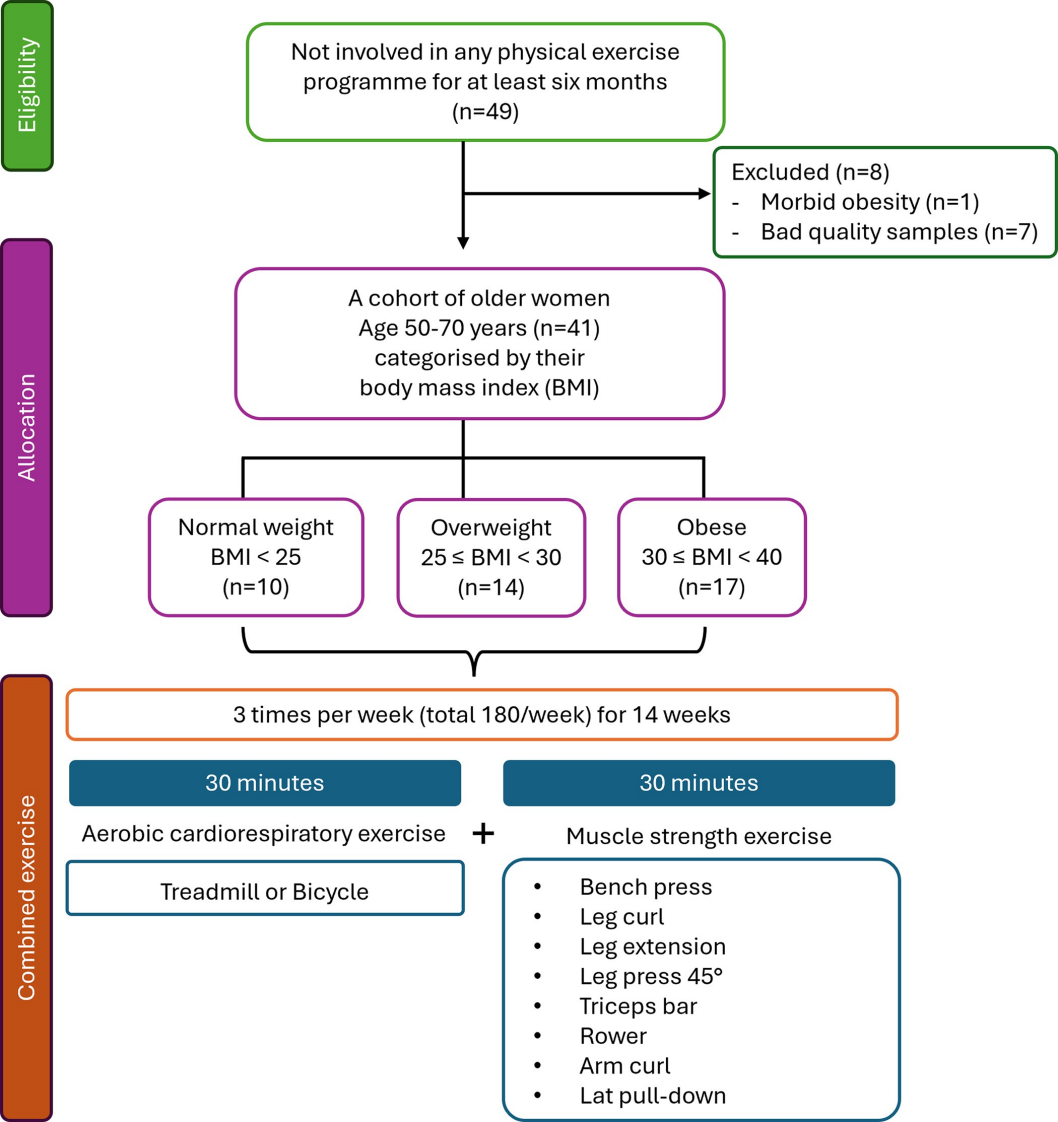
Flow chart of study design.

After providing consent, they all underwent a thorough medical examination including a physical examination by a doctor. This examination also included cognitive assessments and quality of life evaluations (**S1 Table**) to determine their eligibility for engaging in exercise and to ensure they met the study requirements. We also collected the pre-existing health conditions and medications used by participants in **S2 Table**. The inclusion criteria required participants to be able to perform moderate physical activity and to be willing to participate in the 14-week combined exercise program. The exclusion criteria included severe cardiovascular or respiratory diseases, recent major surgery, diagnosed cognitive impairment, or severe psychiatric disorders. Additionally, it was ensured that these participants had not been involved in any physical exercise programme for at least six months. A total of 49 older women were enrolled in this study. We formed a cohort of 42 older women, aged between 50 and 70 years, and categorised them into three distinct groups according to their body mass index (BMI), following the guidelines set forth by WHO [19] and: normal weight (BMI < 25), overweight (25 ≤ BMI < 30), and obese (30 ≤ BMI < 40). One participant (morbid obesity) was excluded due to a BMI ≥ 40 (**Fig 1**).

### Study design

The study design is more comprehensively outlined in da Silva Rodrigues’s study [20]. Briefly, this longitudinal study examined the physical benefits of a 14-week combined exercise programme consisting of 30 minutes of aerobic cardiorespiratory exercises on a treadmill or bicycle, followed by 30 minutes of muscle strength exercises (bench press, leg curl, leg extension, leg press 45°, triceps bar, rower, arm curl, and lat pull-down). The participants performed the training three times a week (totalling 180 minutes per week) for 14 weeks. The periodization adopted for this study involved a progressive approach. During the first two weeks, participants engaged in 30 minutes of aerobic exercise at 50% of their heart rate, accompanied by strength exercises consisting of 15 to 17 maximum repetitions. Subsequently, in the following weeks leading up to the completion of the 14-week programme, participants performed aerobic exercise at 70% of their heart rate reserve and 10 to 12 maximum repetitions for each strength exercise [9]. To track the intensities adopted in our training protocol, we used a heart rate monitor (Polar Team2) throughout the training session. To calculate the heart rate reserve, the following formula was used: % heart rate reserve = target intensity (in decimals) × [(maximum heart rate (MHR)—resting heart rate) + resting heart rate]. The MHR was determined using the formula; MHR = 220—participant’s age, while the resting heart rate was measured after the participant had rested in the supine position for ten minutes.

Anthropometric measurements such as body weight and height were taken to calculate BMI. Body fat percentage (% fat) was assessed using the G-Tech digital scale (model Balgl200). Blood pressure measurements were taken with an automatic digital upper arm monitor (OMRON^®^ model HEM-7113) for both systolic and diastolic pressures. For biochemical assessments, including total cholesterol, HDL, LDL, and triglycerides, plasma from an 18-hour fasting blood sample was analysed using the Wiener Lab BT 3000 Plus auto-analyser. The assessments were conducted at three different time points: baseline (PRE), 7 weeks (POST7), and 14 weeks (POST14) after the start of the training programme. Moreover, physical performance tests were performed before and after exercise training including the Sit-to-Stand test, elbow flexion and extension (EFE), and the aerobic capacity was assessed using a six-minute walk test (6MWT) [21], which measures the walking distance covered in metres within six minutes. Blood DNAm profiles before and after the combined exercise were measured by Infinitum MethylationEPIC BeadChip (850K).

### Data analysis

Methylation profiles were analysed using the Bioconductor package in R Studio (version 4.2.2). Probes with detection *p*-values higher than 0.01 were excluded from the analysis. The data was then normalised using the PreprocessNoob method in the minfi package. To address probe-type bias, the dataset was adjusted for both Infinium I (type I) and Infinium II (type II) probes [22]. Additionally, 43,254 loci with cross-reactive probes and 59 explicit SNP probes ("rs" probes) were removed from the dataset [23]. To address potential batch effects as a technical variable, the ComBat function from the sva package [24] was utilised for detection and correction. Additionally, in order to account for cell-type heterogeneity in blood, the correction was performed using the reference-based RefbaseEWAS method [25] from the ChAMP package. The methylation level of each CpG site was assessed by calculating a β value ranging from 0 to 1, based on the ratio of fluorescent intensities. Differential methylation analysis was conducted using the limma package [26] to identify differentially methylated positions (DMPs) in blood samples from older women. The results from this analysis were subjected to multiple testing corrections using the Benjamini–Hochberg method [27], which controls the false discovery rate (FDR), reducing the likelihood of false positives when dealing with large datasets. DMPs with Benjamini-Hochberg adjusted *p*-values < 0.05 were considered significant.

To explore the biological functions and gene regulatory networks (Kyoto Encyclopaedia of Genes and Genomes (KEGG) pathways and Gene Ontology (GO), a list of significantly altered genes was analysed using pathfindR [28]. An active subnetwork is identified by a group of genes that are interconnected within a protein-protein interaction network. The top 10 significant pathways and GO terms with adjusted *p*-values (by Benjamini–Hochberg (BH) method) < 0.05 were identified. MethylDetectR automates the calculation of estimated values or scores for a variety of human traits as the systematic and reproducible creation of incident disease predictors. According to this analysis, DNAm-based associated with alcohol consumption, body mass index, high-density lipoprotein cholesterol and smoking behaviour was calculated to generate Episcore [29]. β DNAm values of selected probes (**S3 Table**) were uploaded into https://shiny.igc.ed.ac.uk/Calculate_Your_Scores/.

Furthermore, our study incorporated 155 lipogenesis and 138 lipolysis-related genes sourced from the GeneRIF Biological Term Annotations dataset. The database integrated novel functionality of genes from the National Center for Biotechnology Information (NCBI) Gene database(https://maayanlab.cloud/Harmonizome/gene_set/lipogenesis/GeneRIF+Biological+Term+Annotations,https://maayanlab.cloud/Harmonizome/gene_set/enhanced+lipolysis/MPO+Gene-Phenotype+Associations).

### DNA methylation age (DNAm age)

DNA methylation age (DNAm age) was predicted using the new DNA methylation Age Calculator, an open-access tool available at https://dnamage.genetics.ucla.edu/ [30]. This tool provided DNAm age predictions for eight models, including Horvath, Hannum, PhenoAge, Skin and Blood, Zhang, FitAge, GrimAge, and GrimAge2. DNAm age acceleration, an indicator of more rapid ageing and referring to the residuals of the regression of epigenetic age on chronological age, was included in the predicted results. This encompassed Horvath intrinsic epigenetic age acceleration (Horvath IEAA), Hannum IEAA, PhenoAge acceleration (PhenoAA), and GrimAge acceleration (GrimAA).

### Statistical analysis

Given the sample size of fewer than 50 participants, the Shapiro-Wilk test was employed to assess the normality of the data using GraphPad Prism Statistical Software (version 9.0.0). For comparisons of POST7 or POST14 with baseline, the paired sample *t*-test and the Wilcoxon matched-pairs signed-rank test were employed to compare parametric and non-parametric continuous variables, including individual characteristics, DNAm by genomic and CpG density location, lipid-related genes, and predicted protein levels. These analyses were conducted in GraphPad Prism Statistical Software. For the comparison of DNAm levels of lipid-related genes and predicted protein levels among groups, an ANCOVA (conducted in the IBM® SPSS statistical software programme (version 24)) with BMI and age as covariates was conducted to explore differences among normal weight, overweight, and obese groups. The inclusion of these covariates was necessary to account for their potential influence on the outcomes, as both BMI and age are known to affect DNAm patterns and protein expression. The comparison of DNAm age and AgeAcceleration within and between groups was performed using the Kruskal–Wallis rank sum test, followed by Dunn’s test for post-hoc analysis, in RStudio (Version: 2023.06.0–421). Additionally, Spearman’s Rank correlation coefficient was used to examine the correlation between chronological age and DNAm age, also utilising RStudio. Statistical significance was defined as *p*-value < 0.05.

## Results

### Characteristics of participants’ blood pressure, biochemical variables, and improvement of physical performances after combined exercise

To assess the impact of the combined exercise training programme on functional outcomes and whole blood DNAm in older women, we conducted evaluations at baseline, 7 POST7, and POST14 after the start of the combined exercise. The participants’ anthropometric parameters, blood pressure, physical performance, and biochemical measurements were assessed at these time points. A total of 41 participants completed the 14-week combined exercise and were categorised based on BMI into the following groups: normal weight (*n* = 10): BMI < 25, overweight (*n* = 14): 25 ≤ BMI < 30, and obese (*n* = 17): 30 ≤ BMI < 40. This grouping allowed us to examine the effects of combined exercise across different BMI categories.

Firstly, we assessed the clinical and physical parameters of all participants at the 7^th^ and 14^th^ weeks compared to baseline. In the 14^th^ week, blood pressure and lipid profiles, except for HDL, showed improvement following the combined exercise. Physical performance, measured by the sit-to-stand test, EFE, and 6MWT, significantly increased in all participants after the combined exercise at both the 7^th^ and 14^th^ weeks (**S4 Table**). After analysing these parameters based on the BMI category, our study revealed a notable decrease in blood pressure and improvements in lipid profiles, as shown in **Table 1**. Compared to the baseline, systolic blood pressure (SBP) significantly decreased at POST7 and POST14 in both overweight and obese groups. Both groups also showed a substantial decrease in cholesterol levels at POST14, while only the overweight group showed a decrease in triglyceride levels at POST14. Moreover, the BMI of obese participants significantly decreased only at POST7.

**Table 1:** Characteristics of biochemistry, anthropometric and physical performance data of older women before and after combined exercise among normal, overweight and obese groups.

Variable	Normal weight			Overweight			Obese		
	Baseline	7th week	14th week	Baseline	7th week	14th week	Baseline	7th week	14th week
Age (year)	59.7±5.7	59.9±5.7	60.0±5.7	62.5±4.6	62.7±4.6	62.8±4.6	60.2±6.2	60.5±6.2	60.6±6.2
BMI (kg/m^2^)	23.6±1.3	24.5±3.4	25.1±2.6	28.1±1.3	28.1±2.4	27.9±2.1	33.0±2.3	31.3±3.1*	32.1±2.3
SBP (mmHg)	122.7±11.4	114.7±10.7	115.9±9.1	129.4±15.0	122.4±11.6*	120.5±14.0*	138.3±20.4	137.5±16.1	123.3±16.3*
DBP (mmHg)	75.9±9.4	78.6±11.6	73.9±6.7	78.9±6.6	80.1±7.1	75.5±5.4	79.8±12.1	79.3±9.6	74.5±7.6*
Sit-to-stand (rep)	14.8±3.9	17.9±4.8*	18.1±5.6	13.6±2.8	16.5±4.9*	16.4±4.4*	12.5±3.0	17.2±5.3*	17.3±5.2*
EFE (rep)	18.5±4.1	21.9±1.6*	21.3±4.2	17.9±4.5	19.3±2.2	19.5±3.1	18.9±3.6	20.2±3.3	22.6±4.0*
6MWT (mins)	541.5±88.1	567.0±48.2	614.8±69.7*	553.5±57.6	561.8±50.8	551.6±39.3	511.5±54.3	548.2±55.9*	574.6±53.7*
Cholesterol (mg/dL)	209.5±15.2	199.1±41.3	193.7±19.1	213.4±34.3	196.8±29.4	202.3±32.6*	219.2±40.4	209.3±41.1	200.8±47.2*
HDL (mg/dL)	52.8±9.0	56.4±17.1	52.4±9.9	58.4±14.5	51.5±12.1	51.4±12.3	52.2±9.7	52.1±11.1	48.8±9.7*
LDL (mg/dL)	127.9±16.9	118.2±33.9	116.2±23.1	130.9±31.2	122.4±26.4	129.8±28.5	138.0±43.0	131.7±38.6	126.1±46.4
Triglycerides (mg/dL)	143.0±51.6	122.4±36.3	125.3±31.1	120.4±32.3	114.9±34.8	105.6±29.2*	145.1±67.0	127.2±47.5	129.2±47.3
FAT (%)	33.7±7.6	31.2±11.5	32.9±9.5	40.9±4.9	41.4±3.1	37.3±10.0	44.7±8.1	44.1±7.0	36.2±5.2

Data showed as mean ± standard deviation; body mass index (BMI), systolic blood pressure (SBP), diastolic blood pressure (DBP), elbow flexion and extension (EFE), six-minute walk test (6MWT), high-density lipoprotein (HDL), low-density lipoprotein (LDL). * Statistical significance compared to baseline (*p*-value < 0.05).

Regarding physical performance, the Sit-to-Stand test improved significantly at POST7 in normal-weight and overweight groups, remaining consistent throughout the study. EFE improved within 7 weeks in the normal-weight group, while the obese group showed increased EFE performance at both POST7 and POST14 compared with baseline. Additionally, the obese group demonstrated improved performance in 6MWT at POST7 and POST14, while normal-weight group showed this improvement only at POST14.

### The effect of combined exercise on blood DNA methylation profiles of older women with obesity

**Fig 2:**
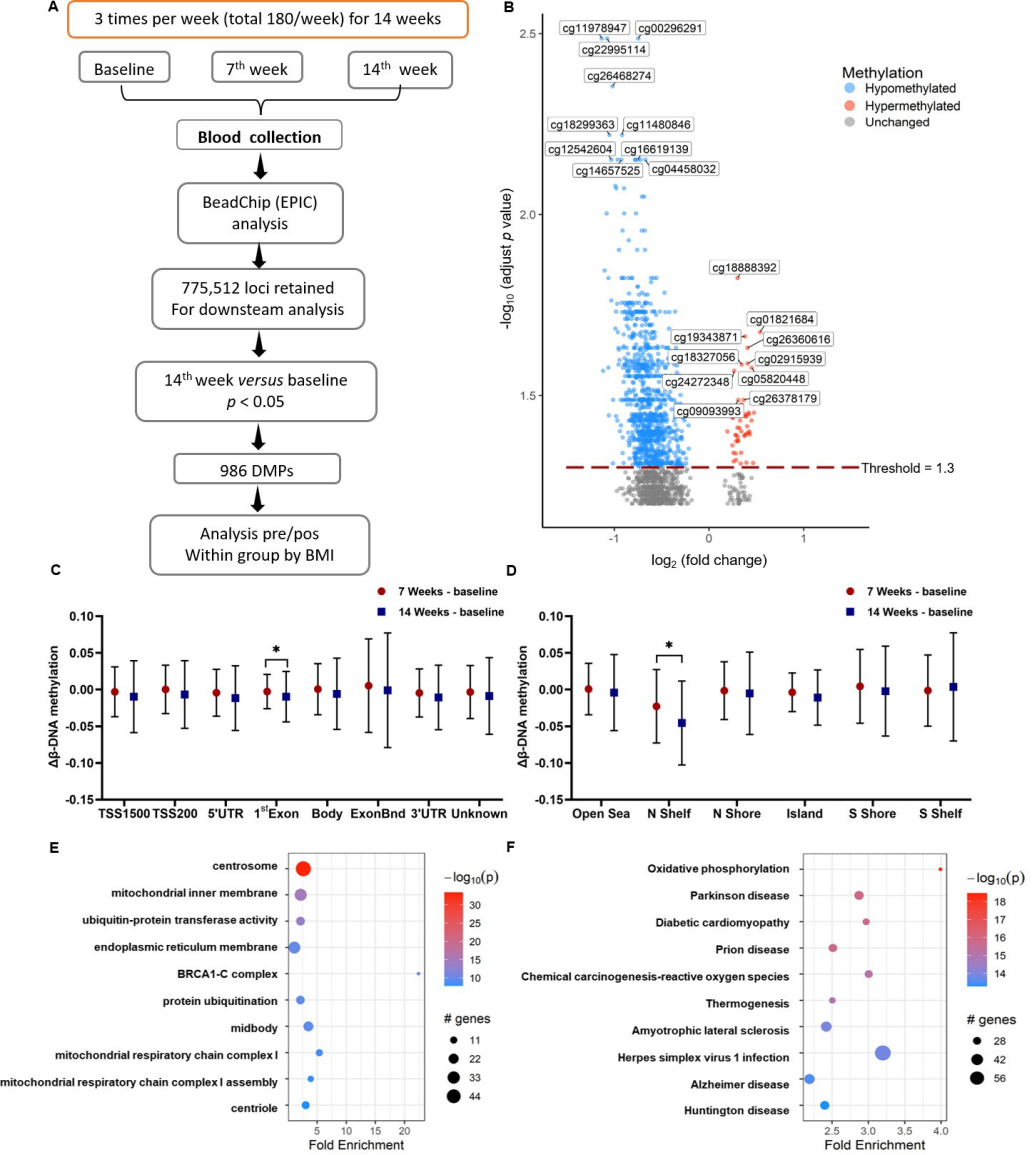
DNA methylation profiling at POST14 after the combined exercise in older women. A) Study design. B) Volcano plots of the differentially methylated positions (DMPs). C-D) Alterations in DNA methylation of DMPs at 7-week and 14-week follow-up by genomic location (C) and CpG density (D). E-F) Pathway and Gene Oncology analysis of the 986 DMPs by Kyoto Encyclopedia of Genes and Genomes (E) and Gene Oncology (F). N Shelf, North Shelf; N shore, North shore; S Shelf, South Shore; S Shelf, South Shelf; TSS1500, 1500 base pairs regions upstream transcription start sites; 5’UTR, The 5′ untranslated region; ExonBnd, within 20 bases of an exon boundary; 3’UTR, The 3′ untranslated region. Asterisk (*) indicates significant differences in Δβ DNA methylation between the 7^th^ week and the 14^th^ week (*p*-value < 0.05).

In our study, the blood DNAm levels of a total of 41 older people were examined at three different time points and the study design is summarised in **Fig 2A**. Initially, we analysed the global DNAm by calculating the average across all CpG sites in the 850K microarray. We found that no statistical significance existed in the global DNAm levels for participants regardless of the BMI group or time (**S1 Fig**). However, the analysis of gene-specific DNAm of all participants at POST14 compared to the baseline revealed a total of 986 significant DMPs, which showed Benjamini-Hochberg adjusted *p*-values < 0.05. These consisted of 939 hypomethylated and 47 hypermethylated DMPs, as depicted in **Fig 2B**. The top 10 hypomethylated and hypermethylated DMPs of all subjects at POST14 are detailed in **Table 2** and **S2, S3 Figs**. We demonstrated a significant decrease in DNAm levels over time across all subjects, comparing the DNAm levels at each genomic location across three different time points. This decrease was found in the North shelf and 1^st^ Exon, as depicted in **Fig 2C, 2D**.

**Table 2:** Top 10 differentially methylated positions after 14 weeks of combined exercise.

CpG	LogFC	Adj.P.Val (by BH method)	CHR	Gene	Genomic location	Relation to island
**Hypomethylated DMPS**						
cg00296291	-0.748	0.003	4	*PHF17*	5’UTR	Island
cg11978947	-1.132	0.003	12	*SNRNP35*	TSS200	N_Shore
cg22995114	-1.076	0.003	2	*ID2*	Gene body	Island
cg26468274	-1.019	0.004	6	*XPO5*	TSS200	Island
cg11480846	-0.918	0.006	9	*CCDC183-AS1*	TSS200	S_Shore
cg18299363	-1.051	0.006	10	*C10orf76*	TSS200	Island
cg04458032	-0.672	0.006	7	*AGFG2*	TSS1500	N_Shore
cg12542604	-1.032	0.007	6	*ANKS1A*	TSS1500	N_Shore
cg14657525	-0.925	0.006	3	*TRIM71*	TSS1500	Island
cg16619139	-0.752	0.006	11	*DGKZ*	TSS1500	OpenSea
**Hypermethylated DMPS**						
cg18888392	0.303	0.014	8	*FAM110B*	5’UTR	OpenSea
cg01821684	0.541	0.021	7	*RAC1*	Gene body	S_Shore
cg19343871	0.381	0.022	19	*BICRA*	Gene body	Island
cg26360616	0.408	0.024	4	*INTU*	Gene body	Island
cg02915939	0.409	0.026	6	*IER3*	3’UTR	Island
cg24272348	0.267	0.026	19	*CD79A*	TSS1500	OpenSea
cg05820448	0.453	0.027	7	*SHH*	TSS200	Island
cg26378179	0.368	0.033	14	*GALC*	Gene body	OpenSea
cg09093993	0.312	0.033	8	*PLEKHF2*	TSS1500	N_Shore
cg18327056	0.347	0.036	2	*KCNK3*	TSS1500	N_Shore

LogFC, log fold change; Adj.P.Val, adjusted *p*-value; BH, Benjamini–Hochberg; CHR, chromosome; N_Shelf, North Shelf; N_shore, North shore; S_Shelf, South Shore; S_Shelf, South Shelf; TSS1500, 1500 base pairs regions upstream transcription start sites; 5’UTR, The 5′ untranslated region; 3’UTR, The 3′ untranslated region.

To extend the understanding of the effects of combined exercise on biological processes and functions, 986 DMPs located on 744 genes were further analysed for GO terms and KEGG pathways. The GO analysis revealed a cluster of genes related to cellular metabolisms, such as centrosome, mitochondrial inner membrane, ubiquitin-protein transferase activity, endoplasmic reticulum membrane, mitochondrial respiratory chain complexes I, mitochondrial respiratory chain complex I assembly, and centriole (Benjamini-Hochberg adjusted *p*-values < 0.05) (**Fig 2E**). Meanwhile, the KEGG pathway enrichment analysis showed a group of genes involved in various pathways, including oxidative phosphorylation, chemical carcinogenesis–reactive oxygen species, thermogenesis, diabetic cardiomyopathy, and Parkinson’s disease (Benjamini-Hochberg adjusted *p*-values < 0.05) (**Fig 2F**).

**Fig 3:**
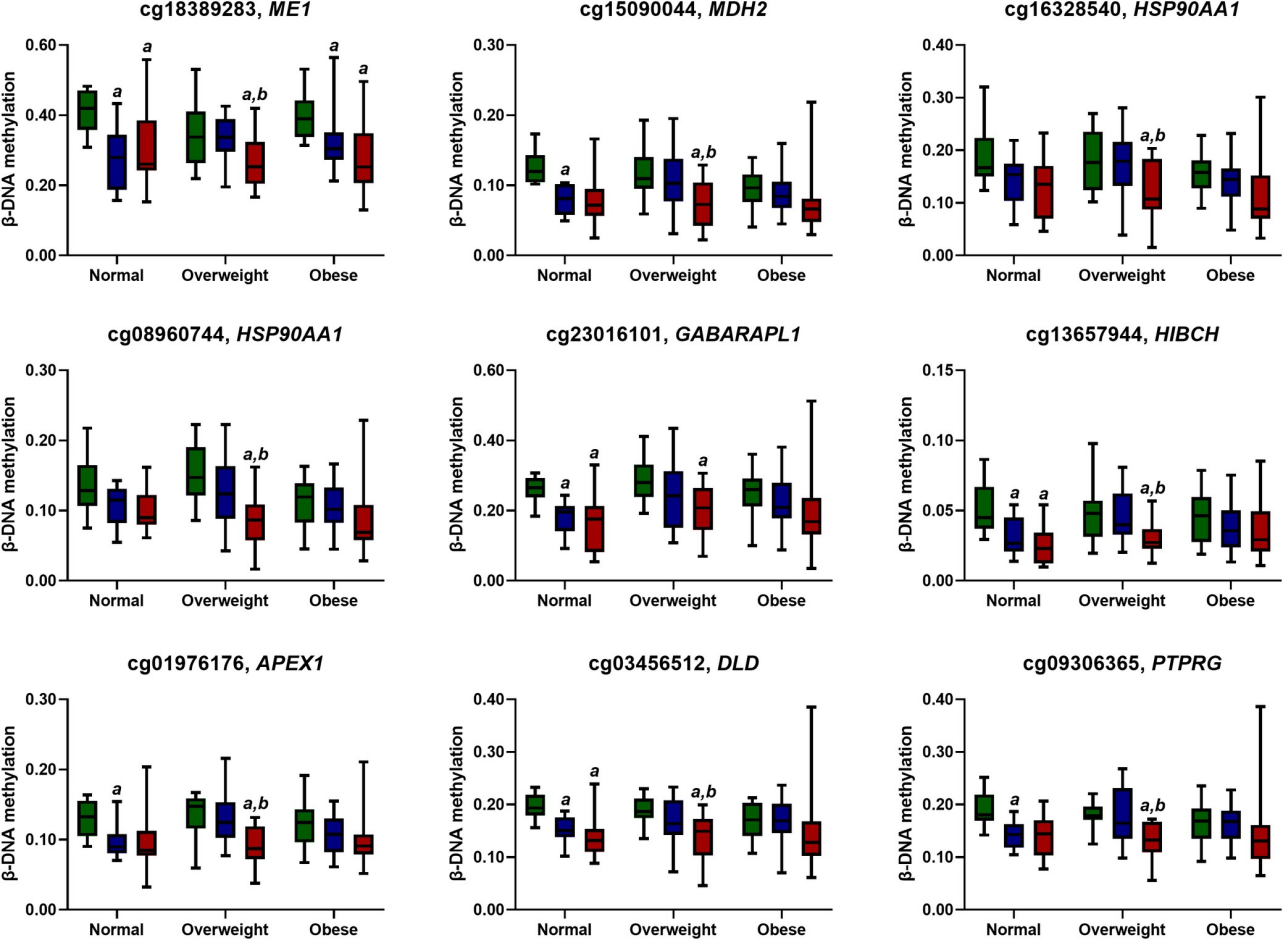
A panel of β-DNA methylation levels of lipid-related genes across BMI groups. The results are presented as Median ± SD. ^a^ significant difference compared to baseline (*p*-value < 0.05). ^b^ significant difference compared to 7-week follow-up (*p*-value < 0.05). The colour coding used is as follows: baseline, green; 7-week follow-up, blue and 14-week follow-up, red.

By exploring the effects of our exercise intervention on DNAm patterns, we aimed to gain deeper insights into the molecular mechanisms underlying the regulation of lipogenesis in response to exercise. We extended our investigation to examine the impact of a combined exercise regimen on DNAm levels at specific gene loci associated with lipogenesis. We employed a list of 155 lipogenesis-related genes and 138 lipolysis-related genes from the GeneRIF Biological Term Annotations dataset. Our analysis revealed that among the nine loci located on eight genes identified. For normal-weight subjects, our results showed a significant decrease in methylation levels at cg18389283 (*ME1*), cg23016101 (*GABARAPL1*), cg13657944 (*HIBCH*), and cg03456512 (*DLD*) at both POST7 and POST14 compared to baseline. However, DNAm levels at cg15090044 (*MDH2*), cg01976176 (*APEX1*), and cg09306365 (*PTPRG*) were only reduced at POST7. Interestingly, overweight subjects showed a substantial reduction in methylation levels at all nine gene loci only at 14 weeks post-exercise. In contrast, the obese group showed a significant decrease only at the cg18389283 (*ME1*) locus at both POST7 and POST14, with no significant differences observed at the other eight loci. These results are summarised in **Fig 3**. Moreover, only the DNAm levels of *APEX1* showed a significant decrease in the obese group at POST7 compared to the overweight group (**S5 Table**).

**Fig 4:**
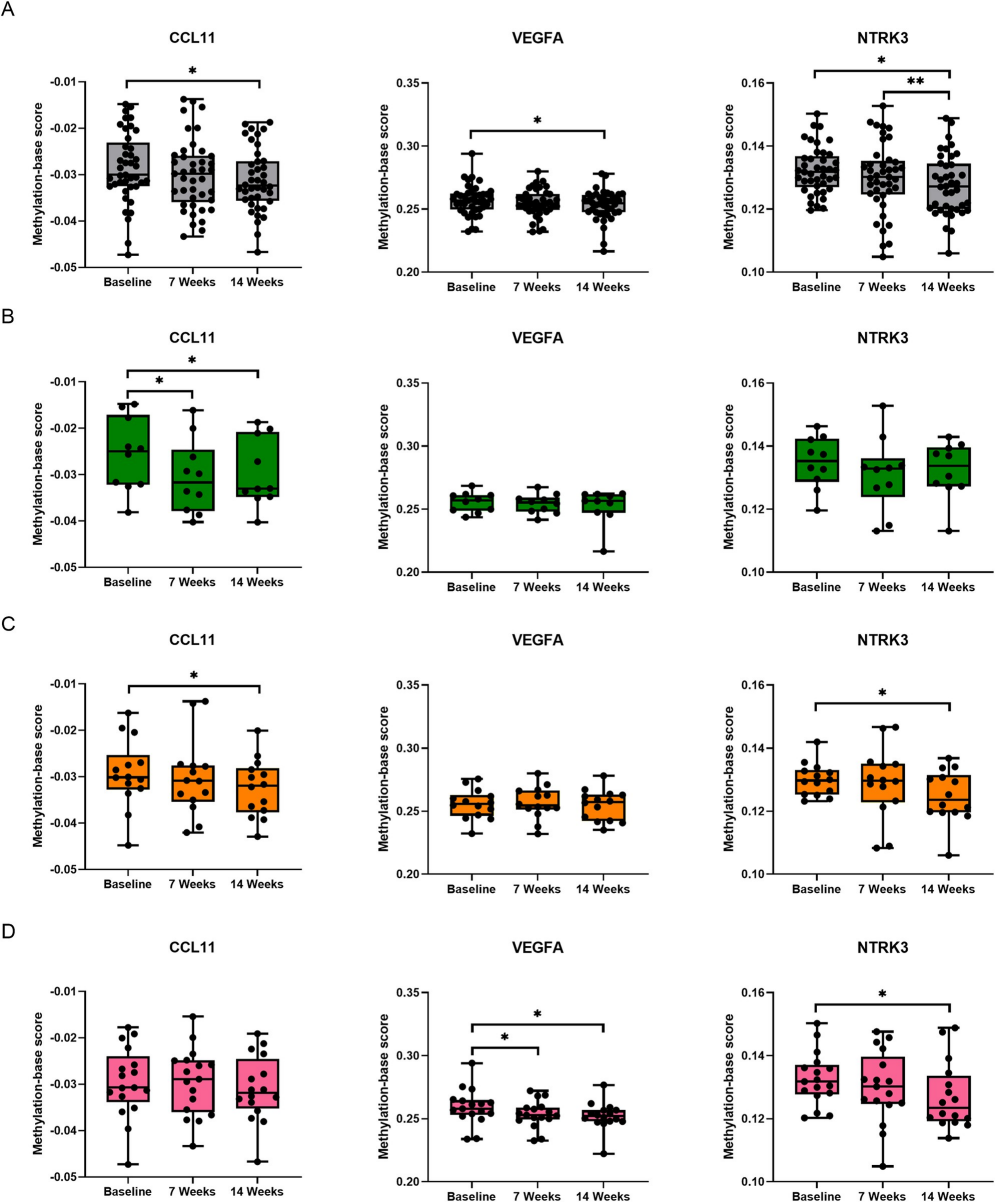
Predicted protein levels by protein EpiScores. A) A panel of CCL11, VEGFA, and NTRK3 levels in all participants at baseline and after the combined exercise at weeks 7 and 14. B-D) A panel of CCL11, VEGFA, and NTRK3 levels across sub-groups by BMI at baseline and after the combined exercise at weeks 7 and 14: normal weight (B), overweight (C), and obese (D). The results are presented as Median ± SD. * significant difference compared to the baseline (*p*-value < 0.05). The colour coding used is as follows: normal weight, green; overweight, orange and obese, pink.

We further explored protein EpiScores, which are DNAm-based proxies used to estimate protein levels including CCL11, VEGFA, and NTRK3. We observed significant reductions in CCL11, VEGFA, and NTRK3 levels at POST14 compared to baseline (**Fig 4A**). Additionally, NTRK3 exhibited a further reduction at POST14 compared to POST7. After sub-analysis by BMI categories, only normal-weight participants demonstrated a significant decrease in CCL11 levels at POST7 and POST14 from -0.026 to -0.031 and -0.029, respectively (**Fig 4B**). For overweight participants, both CCL11 and NTRK3 substantially reduced at POST14 compared to baseline from -0.029 to -0.032 and 0.130 to 0.125 respectively (**Fig 4C**). For obese participants, VEGFA significantly decreased at both POST7 and POST14 compared to baseline from 0.259 to 0.254 and 0.252, respectively (**Fig 4D**). Additionally, NTRK3 was reduced at POST14 from 0.133 to 0.127. However, the comparison of CCL11, VEGFA, and NTRK3 levels across BMI categories at each time point showed no significant differences (**S6 Table**).

### Association of physical activity with DNA methylation age

**Fig 5:**
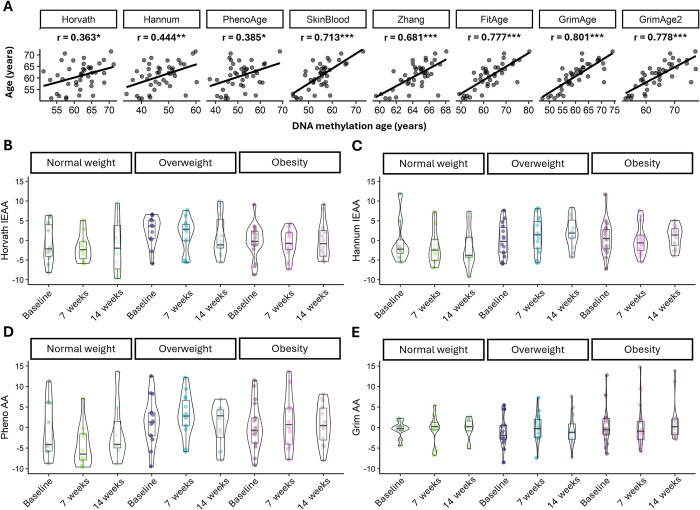
DNA methylation age of older women from blood samples. A) A panel of the correlation between chronological age and DNA methylation (DNAm) age for Horvath, Hannum, PhenoAge, Skin and Blood (SkinBlood), Zhang, FitAge, GrimAge, and GrimAge2. B-E) Age acceleration residuals at baseline, 7 weeks, and 14 weeks after combined exercise using Horvath intrinsic epigenetic age acceleration (Horvath IEAA) (B), Hannum IEAA (C), PhenoAge acceleration (PhenoAA) (D), and GrimAge acceleration (GrimAA) (E). The results are presented as Mean ± SD. * significant difference (*p*-value < 0.05), ** significant difference (*p*-value < 0.01), *** significant difference (*p*-value < 0.001).

In this experiment, we employed eight DNAm age models to examine the effects of combined exercise practices on epigenetic age. At baseline, we observed a correlation between chronological age and DNAm age for all participants: Horvath with r = 0.363 (*p*-value < 0.05), Hannum with r = 0.444 (*p*-value < 0.01), PhenoAge with r = 0.385 (*p*-value < 0.05), SkinBlood with r = 0.713 (*p*-value < 0.001), Zhang with r = 0.713 (*p*-value < 0.001), FitAge with r = 0.777 (*p*-value < 0.001), GrimAge with r = 0.801 (*p*-value < 0.001), and GrimAge2 with r = 0.778 (*p*-value < 0.001) (**Fig 5A**). However, no substantial differences were noted in the age acceleration residual of all three BMI subgroups analysed by Horvath, Hannum, PhenoAge, and GrimAge methods, as illustrated in **Fig 5B–5E**.

## Discussion

Regular physical exercise has been widely recognised as a fundamental component of weight management and overall health improvement. Investigating the effects of exercise on DNAm in the blood of overweight and obese individuals can shed light on the biological mechanisms through which exercise exerts its benefits and potentially identify epigenetic markers associated with exercise-induced health improvements. In this study, we investigated the effects of combined exercise on the epigenome in older women with various BMI categories. Combined exercise, which incorporates both aerobic and strength training exercises within the same session, is recognised to improve aerobic fitness and physical tests such as the 6MWT [31]. The literature supports the effects of combined exercise, highlighting the numerous benefits it offers to the cardiorespiratory system, in the prevention of oxidative stress, and significant metabolic improvements in glycaemic balance [32].

We did not observe significant effects on BMI following the 7^th^ or 14^th^ weeks of combined exercise in overweight and obese. However, we observed a reduction trend in the percentage of body fat in overweight and obese at POST14. This suggests that engaging in strength exercises may lead to muscle gain, which could offset the reduction in fat mass. Due to the higher density of muscle tissue compared to fat tissue, these changes may not be reflected as significant changes in BMI. Furthermore, our findings indicate that combined exercise is effective in improving key functional outcomes among older women, including reductions in SBP and DBP, improvements in the Sit-to-Stand test, enhanced EFE, and increased performance in the 6MWT after 14 weeks of combined exercise. These results align with previous studies where the 6MWT was also significantly improved following fitness intervention [33] or brisk walking [34].

In addition to improving key functional outcomes, several studies have focused on examining the relationship between physical activity and global DNAm levels, as changes in DNAm suggest a potential role for exercise in modulating epigenetic patterns [35]. However, the impact of physical activity on global DNAm levels has generated inconsistent findings across various studies [33, 36, 37]. Some have indicated that exercise does not significantly affect the overall DNAm levels in peripheral blood mononuclear cells, regardless of factors such as age, type of activity, fitness level, or initial DNAm levels [35]. Concomitantly, our findings indicate that there was no significant difference in global DNAm levels either among all blood samples of participants or within subgroups categorised by BMI. Variations in study design, participant characteristics, exercise protocols, and measurement techniques may contribute to the inconsistent findings. Differences in sample types (e.g., blood, muscle, saliva) and DNAm measurement methods (e.g., global quantification methods, genome-wide approaches) can influence the detection of changes in global DNAm. It is important to consider that global DNAm may vary across different tissues and cell types. Thus, our study provides compelling evidence that methylation changes induced by exercise predominantly occur at specific gene loci, rather than affecting global DNAm.

Of all 986 DMPs, the majority of identified DMPs were hypomethylated, with most of these located on TSS1500 (326 DMPs) and CpG Island (325 DMPs). Most of the hypermethylated loci are located on the gene body (13 DMPs) and CpG Island (22 DMPs) (**S4 Fig**). Interestingly, the methylation levels of DMPs located in the 1^st^ Exon and the north shelf significantly decreased at POST14 compared to POST7. Brenet et al. revealed that methylation of the 1^st^ Exon is critical for transcription silencing [38]. Furthermore, the hypermethylated areas such as the North Shelf, South Shelf, and OpenSea are sensitive to external factors (medicines or nutrients), leading to a decrease in DNAm in those areas [39]. Moreover, we observed a time-dependent reduction in DNA methylation, indicating a dynamic process affecting DNAm levels. These DMPs affect genes that regulate cellular metabolism and respiration, which are involved in oxidative phosphorylation (OXPHOS), chemical carcinogenesis–reactive oxygen species, and thermogenesis, according to GO and KEGG pathways analysis. The improvement in these pathways suggests enhanced mitochondrial function and energy metabolism, critical for physical performance and overall metabolic health. Moreover, we identified nine hypomethylated loci located on eight lipid-related genes: *ME1*, *MDH2*, *HSP90AA1*, *GABARAPL1*, *HIBCH*, *APEX1*, *DLD*, and *PTPRG*. These findings offer valuable understanding into the specific molecular mechanisms through which exercise influences lipid metabolism and energy balance. In terms of obesity, the reduction in methylation of lipid-related genes suggests a potential mechanism by which exercise can improve lipid profiles and reduce adiposity. This epigenetic modulation may contribute to the observed reduction in inflammation and cellular oncogenicity, further highlighting the health benefits of regular physical activity.

Generally, the DNAm levels of eight lipid-related genes changed more significantly in normal-weight and overweight women compared to obese women. This difference may be attributed to outliers in DNAm levels from two participants, potentially due to their distinct lifestyle, dietary, and environmental factors, which resulted in greater variability in DNAm patterns. In this study, *ME1* DNAm levels were remarkably decreased in all participants at both POST7 and POST14 after combined exercise. This may be due to an increased cellular energy requirement during intense physical activity [40]. Combined exercise was found to decrease *MDH2* DNAm, particularly in the overweight group. The *MDH2* gene, which encodes an enzyme vital for OXPHOS and the malate-aspartate shuttle, showed increased expression with exercise [40, 41]. Exercise training has been shown to increase *MDH2* in rat cardiac muscle and human skeletal muscles, improving muscle endurance [42, 43]. Moreover, age-related *MDH2* downregulation in human muscles can be reversed by resistance exercise [44]. These findings are consistent with the observed performance improvements in all participants. Our study found that combined exercise led to hypomethylation of *HSP90AA1* and *GABARAPL1*, genes crucial for cellular stress response and adaptation [45–47]. Increased *HSP90AA1* expression was observed in professional taekwondo athletes post-VO_2max_ test [47] and in mice muscles post-exercise [48]. Furthermore, *GABARAPL1* mRNA levels rose by 286% in middle-aged runners after a 200-km race [49]. These trends align with our results, showing significantly reduced DNAm in both *HSP90AA1* and *GABARAPL1* in subjects 14 weeks post-exercise, especially in the overweight group. *HIBCH*, a gene that encodes 3-hydroxyisobutyryl-CoA hydrolase, is an enzyme involved in branch-chain amino acid (BCAA) catabolism. This process yields propionyl-CoA as the final product, which is used for the TCA cycle [50]. In the present study, combined exercise led to a significant reduction in DNAm at *HIBCH* in both normal and overweight participants at POST14. This suggests that the increase in *HIBCH* expression is likely due to enhanced BCAA catabolism to meet energy requirements during exercise [51]. Exercise generates ROS and RNS, causing oxidative damage to lipids, proteins, and DNA [52, 53]. This damage is managed by DNA repair machinery, including *APEX1*, a key endonuclease in the base excision repair (BER) mechanism [54]. Studies have shown increased *APEX1* expression in rats undergoing exercise training [55] and in muscle biopsies of patients after endurance exercise [56]. Our findings reveal a significant decrease in *APEX1* DNAm in normal-weight and overweight participants after combined exercise training, suggesting that increased *APEX1* expression counteracts DNA damage, maintaining cellular homeostasis. *DLD*, encoding dihydrolipoamide dehydrogenase, is a subunit of the mitochondrial pyruvate dehydrogenase complex. This enzyme is vital for energy production, converting pyruvate into acetyl-CoA [57]. Our study found significant reductions in *DLD* methylation levels in both normal and overweight subjects after exercise training at POST7 and POST14. This aligns with prior research that reported an upregulation of pyruvate dehydrogenase in human skeletal muscle during prolonged moderate-intensity exercise, suggesting that exercise enhances cellular adaptation to increased energy demand [58]. Protein tyrosine phosphatase receptor gamma, encoded by the *PTPRG* gene, is a receptor in the receptor-like protein tyrosine phosphatases family with functions such as bicarbonate sensing and vasorelaxation [59]. Our results revealed a substantial decrease in *PTPRG* DNAm levels at POST7 and POST14 for normal and overweight participants, respectively, after combined exercise. This could be attributed to an increase in metabolic acidosis due to excessive proton accumulation during ATP breakdown induced by intense exercise [60].

EpiScores-based protein level estimation revealed a marked decrease in CCL11, VEGFA, and NTRK3 levels in subjects at 14 weeks post-exercise relative to baseline. CCL11, known as eotaxin-1, serves as an immunomodulator, attracting various immune cells, including macrophages, neutrophils, basophils, eosinophils, mast cells, and type 2 helper T-lymphocytes [61]. Elevated CCL11 levels are typically observed in individuals with chronic inflammatory diseases [62]. Additionally, increased plasma CCL11 levels have been associated with ageing in mice and humans and have been shown to inhibit neurogenesis in mice, thus impacting the nervous system negatively [63]. Previous studies have indicated that aerobic exercise training notably reduced plasma eotaxin-1 levels in obese young men compared to baseline [64]. Moreover, a consistent 12-week regimen of aerobic and resistance exercise training has been found to significantly decrease levels of fractalkine, TGF-β1, eotaxin-1, and IL-6 in young and middle-aged adults with mobility impairments [65]. Our study also demonstrated a significant reduction in VEGFA levels at both POST7 and POST14 in only the obese group. VEGFA primarily facilitates angiogenesis and exacerbates inflammation by increasing vascular permeability and promoting macrophage and granulocyte migration [66]. Elevated VEGFA levels have been linked to granulomatous inflammation observed in conditions such as tuberculosis, sarcoidosis, and inflammatory bowel disease [67]. Research by Gunga *et al*. showed a significant decrease in circulating VEGFA levels in participants following a high-altitude marathon run [68]. However, some studies have reported an increase in VEGF levels in skeletal muscles after acute exercise training [69, 70], likely due to acute exercise inducing VEGF expression in response to stress adaptation, thereby enhancing oxygen supply to muscle cells through angiogenesis [71]. Tropomyosin receptor kinase (*NTRK3*) is a transmembrane protein encoded by the proto-oncogenic *NTRK3* gene, binding neurotrophins to regulate neuronal development, differentiation, and survival [72]. However, research indicates *NTRK3* can also have oncogenic effects when fused with *TPM3*, promoting cell growth and resistance to apoptosis, leading to neuronal and extra-neuronal neoplasms [73]. In our study, overweight and obese participants showed a significant reduction in NTRK3 levels after 14 weeks of combined exercise. Several studies have established a link between exercise and cancer prevention [74, 75]. For instance, a randomized controlled trial demonstrated a decrease in Ki67-positive cells in colonic crypts after 12 months of moderate-to-vigorous aerobic exercise [76], suggesting chronic exercise may have an anti-cancer effect by regulating the expression of oncogenic markers.

Engaging in high levels of physical activity during old age has been associated with improved health outcomes, an extended life span, and enhanced quality of life [77]. However, the influence of physical activity on life span remains uncertain when genetic factors are taken into account. A better understanding of the interplay between physical activity, genetic factors, and life span can provide valuable insights into the complex relationship between lifestyle choices and longevity. Here, we identified a fair correspondence between DNAm age and chronological age (positive correlation) at the baseline of all participants, with the strongest correspondence observed in the GrimAge model, which outperforms all other DNAm-based biomarkers across various health-related metrics [78]. Furthermore, at the baseline, our observations revealed that overweight and obese older women exhibited higher age acceleration residuals compared to those with a normal weight. This finding suggests that excess weight may contribute to accelerated ageing processes. Moreover, supporting evidence comes from the analysis of liver tissues, for which it was concluded that age acceleration was more pronounced in obese individuals [79]. These findings collectively highlight the potential impact of weight status on age-related changes in different tissues, underscoring the importance of maintaining a healthy weight for healthy ageing. Recently, in a study by Jokai *et al*., it was observed that females with high fitness levels had, on average, a 1.5-year lower age acceleration compared to a lower fitness level group [80]. This suggests that maintaining a good level of fitness may have a beneficial effect on the ageing process, potentially leading to slower biological ageing. However, we did not observe significant changes in DNAm age after the combined exercise. First, the 14-week duration may have been insufficient to induce measurable changes in biological ageing, particularly considering the relatively low endurance levels of the participants, which may not have stimulated enough physiological stress to drive such modifications. Additionally, other confounding variables such as participants’ diet [81], medication use, or lifestyle changes during the study period might have impacted DNAm patterns [82], masking potential age-related changes. These factors can influence methylation status independent of exercise and contribute to variability in outcomes. Therefore, while exercise is known to modulate DNAm, its impact on biological age may require a more sustained or intense intervention to become evident, and a more controlled environment to isolate its effects from other influences.

Lastly, our study has limitations that should be acknowledged. Firstly, the number of participants in this study was relatively small, which may limit the generalisability of the findings. Additionally, the study focused on a specific population, namely older women with varying BMI, which may restrict the applicability of the results to other demographic groups. Additionally, the use of peripheral blood samples as a proxy for tissue-specific DNA methylation changes may not fully capture the epigenetic modifications occurring in other relevant tissues, such as muscle or adipose tissue. Moreover, the study did not account for potential confounding variables such as dietary habits, medication use, and genetic predispositions, which could influence the observed epigenetic changes and their association with exercise. Furthermore, the study design was a longitudinal study of short duration, which prevents us from establishing a causal relationship between exercise and the ageing process. Therefore, it is crucial to conduct larger-scale studies with diverse populations and longer durations to validate and extend our findings. These future independent studies should incorporate objective measures of physical activity, consider tissue-specific analyses, and control for potential confounding factors to provide a more robust evidence base and enable a better understanding of the specific mechanisms through which exercise influences the ageing process.

## Conclusions

In summary, this study explored the impact of a 14-week combined exercise regimen on functional outcomes, biochemical markers, and DNAm levels in older women. The exercise intervention resulted in significant improvements in blood pressure, lipid profiles, and physical performance, with notable variations across different BMI categories. Overweight and obese participants exhibited decreases in systolic blood pressure and cholesterol levels, while improvements in physical performance were observed in all groups, particularly in the obese group, which showed consistent gains in tests such as the sit-to-stand and 6MWT.

At the molecular level, no significant global DNAm changes were observed, but gene-specific DNAm analysis revealed 986 DMPs post-exercise, with a majority being hypomethylated. These DMPs were enriched in pathways related to cellular metabolism, oxidative phosphorylation, and mitochondrial function, highlighting potential mechanistic links between exercise and metabolic health. Specifically, we identified hypomethylation in eight genes related to lipid metabolism and cellular adaptation to exercise, including *ME1*, *MDH2*, *HSP90AA1*, *GABARAPL1*, *HIBCH*, *APEX1*, *DLD*, and *PTPRG*. These changes could contribute to improved lipid profiles, enhanced energy metabolism, and better cellular stress responses, which are crucial for overall metabolic health. Moreover, our study demonstrated that continuous exercise might help reduce inflammation, as indicated by decreased levels of inflammatory markers such as CCL11, and potentially lower cellular oncogenicity through reductions in oncogene-associated markers like VEGFA and NTRK3. These findings align with the broader literature suggesting that exercise has anti-inflammatory and anti-cancer effects.

Our observations also highlighted an association between excess weight and accelerated ageing processes, evidenced by higher age acceleration residuals in overweight and obese older women compared to their normal-weight counterparts. This underscores the importance of maintaining a healthy weight to support healthy ageing. However, we did not observe significant changes in DNAm age after the combined exercise, indicating that the relationship between exercise and biological ageing requires further exploration. To validate and expand upon these findings, larger-scale studies involving diverse populations and longer durations are necessary. Future research should also incorporate more detailed analyses, including tissue-specific DNAm changes and comprehensive control for potential confounding factors such as diet, medication use, and genetic predispositions. Such studies will provide a deeper understanding of the mechanisms underlying the effects of exercise on the ageing process and help to establish more robust evidence for the health benefits of regular physical activity.

## Supporting information

S1 Fig Global DNA methylation levels at baseline, 7^th^ week, and 14^th^ week after the combined exercise of A) All participants, B) Normal weight group, C) Overweight group, and D) Obese group.
(TIF)

S2 Fig Alteration in DNA methylation of the top ten differentially hypomethylated positions for all participants at baseline, 7^th^ week, and 14^th^ week after the combined exercise. (TIF)

S3 Fig Alteration in DNA methylation of the top ten differentially hypermethylated positions for all participants at baseline, 7^th^ week, and 14^th^ week after the combined exercise. (TIF)

S4 Fig Numbers of differentially methylated positions (DMPs) by A) CpG density and B) Genomic location.
(TIF)

S1 Table Comparisons of global health assessment among the normal weight, overweight, and obese older women at baseline, adjusted for age and BMI. (PDF)

S2 Table Sociodemographic and health characteristics of all participants. (PDF)

S3 Table Probe IDs for protein EpiScore. (XLSX)

S4 Table Comparisons of biochemical, anthropometric and physical performance data of older women at baseline, the 7^th^ week, and the 14^th^ week of the study. (PDF)

S5 Table Comparisons of each DNA methylation level of older women at baseline, the 7^th^ week, and the 14^th^ week of the study. (PDF)

S6 Table Comparisons of estimated protein levels of older women at baseline, the 7^th^ week, and the 14^th^ week of the study. (PDF)
