# Supplementary figures and images for: Impact of combined exercise on blood DNA methylation and physical health in older women with obesity
Source: PLoS One. 2024 Dec 16;19(12):e0315250. doi: 10.1371/journal.pone.0315250 (PMC11649090; doi:10.1371/journal.pone.0315250)

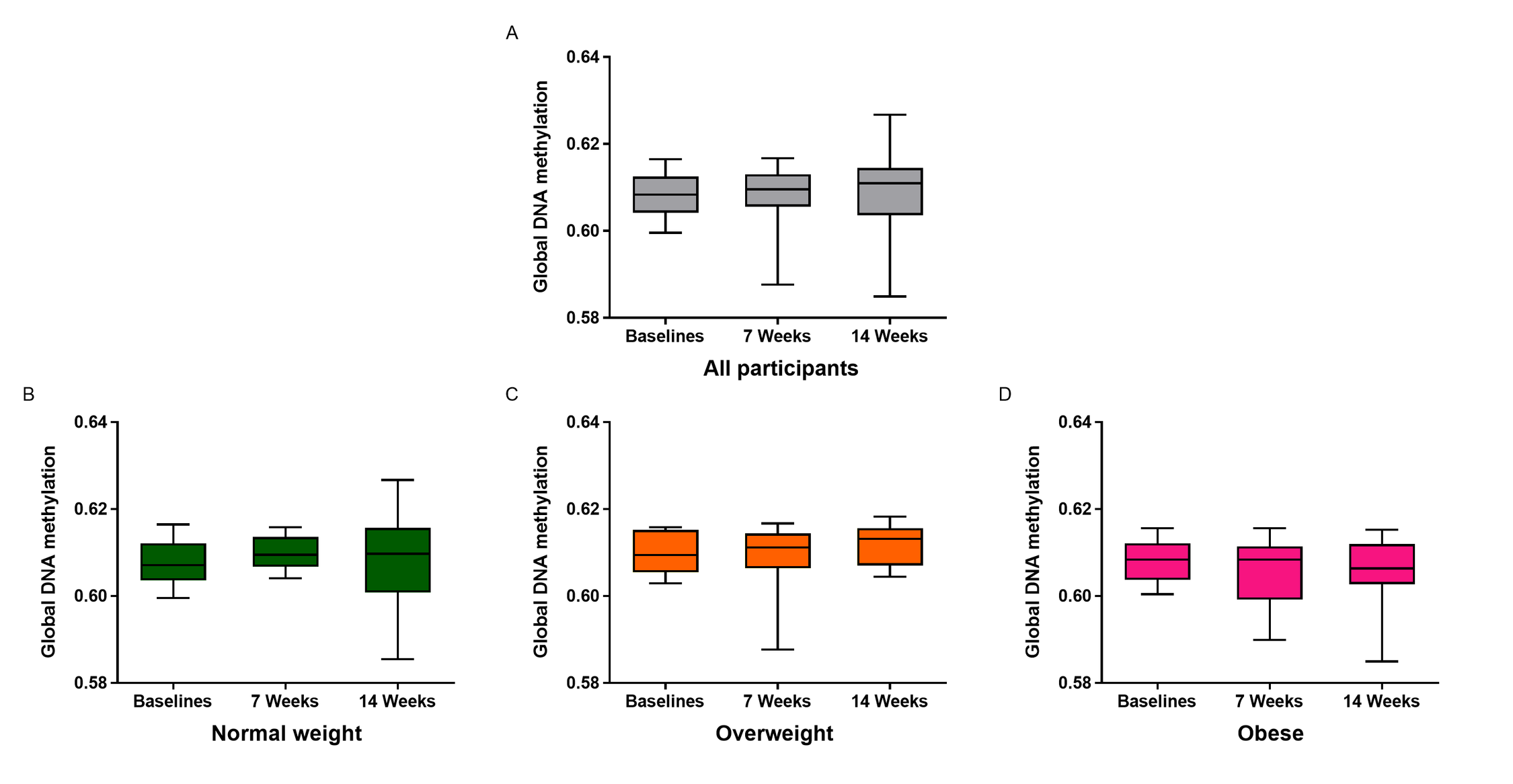

Supplement: S1 Fig — Global DNA methylation levels at baseline, 7th week, and 14th week after the combined exercise of A) All participants, B) Normal weight group, C) Overweight group, and D) Obese group. (TIF) [file pone.0315250.s001.tif]

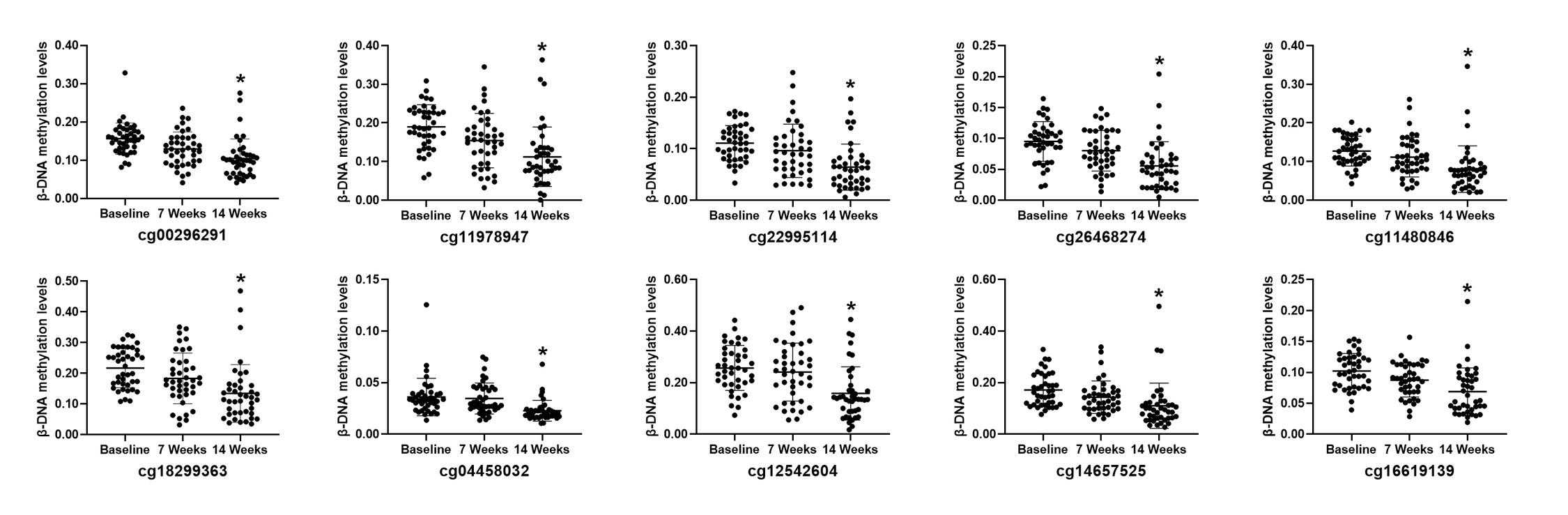

Supplement: S2 Fig — (TIF) [file pone.0315250.s002.tif]

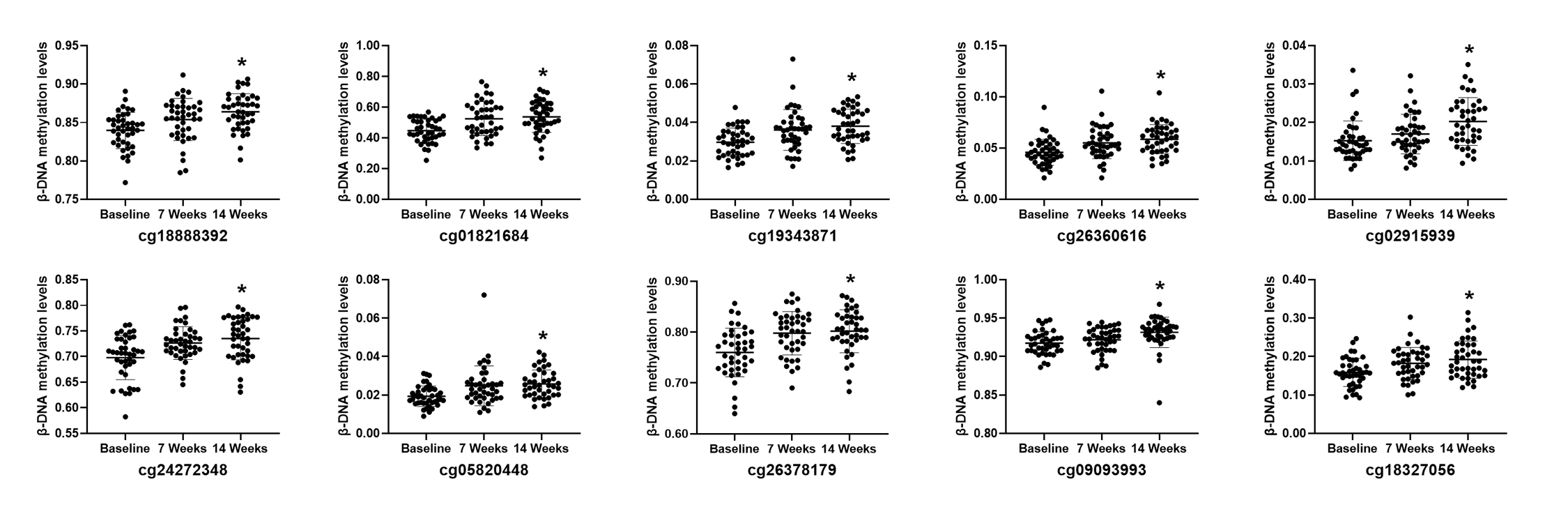

Supplement: S3 Fig — (TIF) [file pone.0315250.s003.tif]

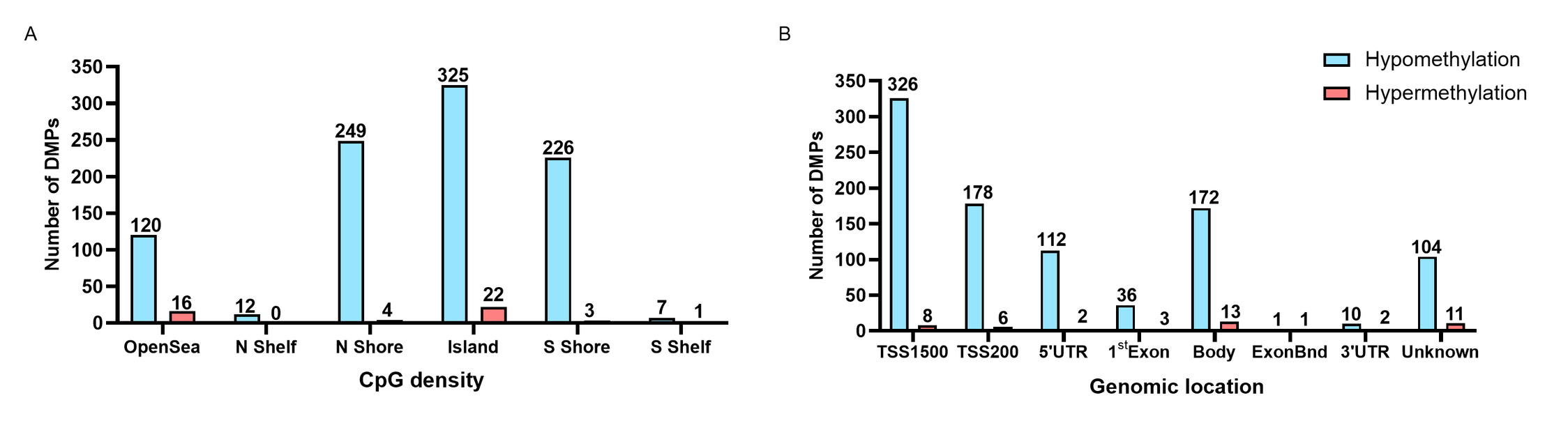

Supplement: S4 Fig — Numbers of differentially methylated positions (DMPs) by A) CpG density and B) Genomic location. (TIF) [file pone.0315250.s004.tif]
