# Supplementary material for: Impact of combined exercise on blood DNA methylation and physical health in older women with obesity
Source: PLoS One. 2024 Dec 16;19(12):e0315250. doi: 10.1371/journal.pone.0315250 (PMC11649090; doi:10.1371/journal.pone.0315250)
Supplement: S1 Table — (PDF) [file pone.0315250.s005.pdf]

S1 Table. Comparisons of global health assessment among the normal weight, overweight, and obese older women at baseline, adjusted for age and BMI.

Global health assessment	Estimated mean ± SE			*p*-value		
	Normal weight (NW)	Overweight (OV)	Obese (OB)	NW vs OV	NW vs OB	OV vs OB
MoCA (score)	22.32±2.50	24.67±1.21	18.62±1.87	0.35	0.37	0.02
GDS (score)	5.19±2.24	3.94±1.09	1.76±1.68	0.58	0.35	0.34
BAI (score)	3.00±3.48	5.87±1.69	4.05±2.60	0.41	0.85	0.60
QBMI (score)	5.47±2.56	3.99±1.24	5.23±1.91	0.56	0.96	0.63
Food consumption markers (score)	24.54±6.00	21.41±2.89	13.64±4.46	0.60	0.27	0.20
SF-36 mental domain (score)	58.45±8.61	54.34±4.18	49.05±6.44	0.63	0.50	0.54
SF-36 physical domain (score)	67.60±7.86	63.89±3.82	58.39±5.88	0.64	0.47	0.49

The data are presented as estimated mean ± standard error (SE) and were analyzed using ANCOVA, with BMI and age as covariates. Significant differences are defined as having a p-value < 0.05. MoCA, Montreal cognitive assessment; GDS, geriatric depression scale; BAI, Beck anxiety inventory; QBMI, modified Baecke questionnaire for the elderly; SF-36, short-form health survey.
