# Supplementary material for: Impact of combined exercise on blood DNA methylation and physical health in older women with obesity
Source: PLoS One. 2024 Dec 16;19(12):e0315250. doi: 10.1371/journal.pone.0315250 (PMC11649090; doi:10.1371/journal.pone.0315250)
Supplement: S2 Table — (PDF) [file pone.0315250.s006.pdf]

**S2 Table.** Sociodemographic and health characteristics of all participants.

| Sociodemographic and health characteristics | Groups (n, %) | | | Total (n = 41) |
|---|---|---|---|---|
| | **Normal weight** (n=10) | **Overweight** (n=14) | **Obese** (n=17) | |
| Marital status | | | | |
| Single | 2 (20.0%) | 1 (7.1%) | 1 (5.9%) | 4 (9.8%) |
| Married | 4 (40.0%) | 8 (57.1%) | 10 (58.8%) | 22 (53.7%) |
| Separated | 3 (30.0%) | 3 (21.4%) | 2 (11.8%) | 8 (19.5%) |
| Widow | 1 (10.0%) | 2 (14.2%) | 4 (23.5%) | 7 (17.1%) |
| Religion | | | | |
| Catholic | 5 (50.0%) | 10 (71.4%) | 15 (88.2%) | 30 (73.2%) |
| Evangelical | 2 (20.0%) | 1 (7.1%) | 1 (5.9%) | 4 (9.8%) |
| Spiritism | 0 | 1 (7.1%) | 1 (5.9%) | 2 (4.9%) |
| Other | 3 (30.0%) | 2 (14.3%) | 0 | 5 (12.2%) |
| Hypertension | | | | |
| Yes | 4 (25.0%) | 6 (42.9%) | 6 (35.3%) | 16 (39.0%) |
| No | 6 (75.0%) | 8 (57.1%) | 11 (64.7%) | 25 (61.0%) |
| Take medication | | | | |
| Yes | 9 (90.0%) | 6 (42.9%) | 10 (58.8%) | 25 (61.0%) |
| No | 1 (10.0%) | 8 (57.1%) | 7 (41.2%) | 16 (39.0%) |
| Smoking | | | | |
| Yes | 0 | 6 (42.9%) | 2 (11.8%) | 8 (19.5%) |
| No | 10 (100%) | 8 (57.1%) | 15 (88.2%) | 33 (80.5%) |
| Diseases | | | | |
| Yes | 8 (80.0%) | 10 (71.4%) | 9 (52.9%) | 27 (65.9%) |
| No | 2 (20.0%) | 4 (28.6%) | 8 (47.1%) | 14 (34.1%) |
