# Supplementary material for: Impact of combined exercise on blood DNA methylation and physical health in older women with obesity
Source: PLoS One. 2024 Dec 16;19(12):e0315250. doi: 10.1371/journal.pone.0315250 (PMC11649090; doi:10.1371/journal.pone.0315250)
Supplement: S4 Table — (PDF) [file pone.0315250.s008.pdf]

**S4 Table.** Comparisons of biochemical, anthropometric and physical performance data of older women at baseline, the 7th week, and the 14th week of the study.

| Variables | **All participants** (n = 41) | | |
|---|---|---|---|
| | **Baseline** | **7th week** | **14th week** |
| BMI ($kg/m^2$) | 29.0±4.1 | 28.6±4.0 | 29±3.6 |
| SBP (mmHg) | 131.4±17.6 | 126.6±16.2* | 120.6±14* |
| DBP (mmHg) | 78.5±9.8 | 79.4±9.1 | 74.7±6.6* |
| Sit-to-stand (rep) | 13.5±3.2 | 17.1±4.9* | 17.2±4.9* |
| EFE (rep) | 18.4±4.0 | 20.3±2.7* | 21.3±3.9* |
| 6MWT (mins) | 533.2±66.1 | 557.8±51.4* | 576.2±57.4* |
| Cholesterol (mg/dL) | 214.8±33.2 | 202.8±37.1* | 199.7±36.5* |
| HDL (mg/dL) | 54.5±11.5 | 52.9±12.8 | 50.6±10.6* |
| LDL (mg/dL) | 133.1±33.7 | 125.5±33.5 | 125.1±35.6* |
| Triglycerides (mg/dL) | 136.3±53.6 | 122±40.5 | 119.8±38.7* |
| FAT (%) | 40.6±8.1 | 40.1±8.8 | 37.1±9.1* |

Data showed as mean ± standard deviation; body mass index (BMI), systolic blood pressure (SBP), diastolic blood pressure (DBP), elbow flexion and extension (EFE), six-minute walk test (6MWT), high-density lipoprotein (HDL), low-density lipoprotein (LDL).
* Statistical significance compared to baseline (*p*-value < 0.05).
