# Supplementary material for: Impact of combined exercise on blood DNA methylation and physical health in older women with obesity
Source: PLoS One. 2024 Dec 16;19(12):e0315250. doi: 10.1371/journal.pone.0315250 (PMC11649090; doi:10.1371/journal.pone.0315250)
Supplement: S5 Table — (PDF) [file pone.0315250.s009.pdf]

S5 Table. Comparisons of each DNA methylation level of older women at baseline, the 7th week, and the 14th week of the study.

Genes	Estimated mean ± SE			*p*-value		
	Normal weight (NW)	Overweight (OV)	Obese (OB)	NW vs OV	NW vs OB	OV vs OB
***ME*1**						
Baseline	0.443±0.043	0.347±0.021	0.374±0.032	0.029	0.325	0.523
7th week	0.278±0.034	0.331±0.024	0.331±0.026	0.210	0.288	0.995
14th week	0.331±0.042	0.271±0.029	0.26±0.034	0.229	0.279	0.807
MDH2						
Baseline	0.097±0.018	0.115±0.009	0.115±0.013	0.306	0.542	0.973
7th week	0.088±0.014	0.108±0.01	0.084±0.011	0.267	0.849	0.128
14th week	0.096±0.014	0.079±0.01	0.053±0.011	0.301	0.054	0.109
HSP90AA1						
Baseline	0.169±0.029	0.174±0.014	0.17±0.022	0.871	0.986	0.893
7th week	0.157±0.022	0.175±0.016	0.134±0.017	0.500	0.493	0.094
14th week	0.142±0.025	0.124±0.017	0.097±0.02	0.539	0.239	0.324
HSP90AA1						
Baseline	0.099±0.023	0.147±0.011	0.14±0.017	0.042	0.277	0.761
7th week	0.113±0.016	0.126±0.012	0.098±0.012	0.510	0.538	0.116
14th week	0.11±0.017	0.087±0.012	0.081±0.014	0.264	0.280	0.738
GABARAPL1						
Baseline	0.288±0.035	0.296±0.017	0.223±0.026	0.800	0.258	0.042
7th week	0.191±0.033	0.24±0.023	0.218±0.025	0.231	0.576	0.528
14th week	0.196±0.038	0.212±0.026	0.162±0.03	0.716	0.558	0.245
HIBCH						
Baseline	0.049±0.012	0.047±0.006	0.048±0.009	0.864	0.967	0.918
7th week	0.031±0.007	0.043±0.005	0.038±0.005	0.159	0.535	0.424
14th week	0.028±0.007	0.032±0.005	0.032±0.006	0.656	0.709	0.958
APEX1						
Baseline	0.12±0.018	0.136±0.009	0.126±0.013	0.372	0.843	0.569
7th week	0.109±0.013	0.133±0.009	0.096±0.01	0.130	0.467	0.009
14th week	0.111±0.015	0.096±0.01	0.085±0.012	0.381	0.259	0.514
DLD						
Baseline	0.196±0.019	0.189±0.009	0.169±0.014	0.708	0.360	0.268
7th week	0.154±0.017	0.168±0.012	0.171±0.013	0.488	0.484	0.879
14th week	0.151±0.02	0.15±0.014	0.128±0.016	0.981	0.459	0.326
PTPRG						

**Genes**	**Estimated mean ± SE**			***p*-value**		
	Normal weight (NW)	Overweight (OV)	Obese (OB)	NW vs OV	NW vs OB	OV vs OB
Baseline	0.175±0.02	0.178±0.01	0.174±0.015	0.881	0.969	0.833
7th week	0.152±0.017	0.176±0.012	0.157±0.013	0.266	0.832	0.319
14th week	0.15±0.02	0.139±0.013	0.125±0.015	0.619	0.400	0.520

The data are presented as estimated mean ± standard error (SE) and were analysed using ANCOVA, with BMI and age as covariates. Significant differences are defined as having a p-value < 0.05.
