# Supplementary material for: Impact of combined exercise on blood DNA methylation and physical health in older women with obesity
Source: PLoS One. 2024 Dec 16;19(12):e0315250. doi: 10.1371/journal.pone.0315250 (PMC11649090; doi:10.1371/journal.pone.0315250)
Supplement: S6 Table — (PDF) [file pone.0315250.s010.pdf]

S6 Table. Comparisons of estimated protein levels of older women at baseline, the 7th week, and the 14th week of the study.

Protein	Estimated mean ± SE			*p*-value		
	Normal weight (NW)	Overweight (OV)	Obese (OB)	NW vs OV	NW vs OB	OV vs OB
**CCL11**						
Baseline	-0.026±0.005	-0.029±0.002	-0.029±0.003	0.428	0.616	0.982
7th week	-0.029±0.003	-0.029±0.002	-0.030±0.002	0.952	0.844	0.843
14th week	-0.026±0.003	-0.032±0.002	-0.033±0.002	0.085	0.115	0.698
**VEGFA**						
Baseline	0.262±0.007	0.256±0.003	0.255±0.005	0.422	0.530	0.826
7th week	0.257±0.004	0.256±0.003	0.251±0.003	0.783	0.346	0.335
14th week	0.254±0.005	0.255±0.004	0.251±0.004	0.914	0.750	0.589
**NTRK3**						
Baseline	0.135±0.004	0.130±0.002	0.132±0.003	0.256	0.613	0.750
7th week	0.128±0.005	0.129±0.003	0.132±0.004	0.879	0.579	0.564
14th week	0.132±0.004	0.125±0.003	0.126±0.003	0.119	0.289	0.875

The data are presented as estimated mean ± standard error (SE) and were analysed using ANCOVA, with BMI and age as covariates. Significant differences are defined as having a p-value < 0.05.
